# Invasive Surgery Impairs the Regulatory Function of Human CD56^bright^ Natural Killer Cells in Response to *Staphylococcus aureus*. Suppression of Interferon-γ Synthesis

**DOI:** 10.1371/journal.pone.0130155

**Published:** 2015-06-19

**Authors:** Renate Reinhardt, Stephanie Pohlmann, Holger Kleinertz, Monika Hepner-Schefczyk, Andreas Paul, Stefanie B. Flohé

**Affiliations:** 1 Department of General-, Visceral- and Transplantation Surgery, University Hospital Essen, University Duisburg-Essen, Essen, Germany; 2 Surgical Research, Department of Trauma Surgery, University Hospital Essen, University Duisburg-Essen, Essen, Germany; University of Sydney, AUSTRALIA

## Abstract

Major surgery increases the risk for infectious complications due to the development of immunosuppression. CD56^bright^ NK cells play a key role in the defense against bacterial infections through the release of Interferon (IFN) γ upon stimulation with monocyte-derived Interleukin (IL) 12. We investigated whether invasive visceral surgery interferes with the IFN-γ synthesis of human NK cells in response to *Staphylococcus aureus*. In a prospective pilot study, peripheral blood mononuclear cells (PBMC) were isolated from 53 patients before and 1 to 7 d after elective visceral surgery. The release of IL-12 and IFN-γ from PBMC upon exposure to *S*. *aureus in vitro* was quantified. The expression of the IL-12 receptor β1 chain on the surface, the phosphorylation of signal transducer and activator of transcription (STAT) 4, and the synthesis of IFN-γ on/in individual CD56^bright^ NK cells were investigated using flow cytometry. The modulatory effect of IL-12 on the *S*. *aureus*-induced IFN-γ production in CD56^bright^ NK cells was analyzed. The IFN-γ secretion from purified CD56^bright^ NK cells was quantified after stimulation with IL-12 and IL-18. After surgery, CD56^bright^ NK cells among total PBMC were impaired in the release of IFN-γ for at least 5 d. Likewise, the IL-12-induced release of IFN-γ from purified CD56^bright^ NK cells was abolished. Upon stimulation with *S*. *aureus*, PBMC secreted less IL-12 but supplementation with recombinant IL-12 did not restore the capacity of CD56^bright^ NK cells to produce IFN-γ. CD56^bright^ NK cells displayed reduced levels of the IL-12Rβ1 chain whereas the phosphorylation of STAT4, the key transcription factor for the *Ifng* gene was not diminished. In summary, after invasive visceral surgery, CD56^bright^ NK cells are impaired in *S*. *aureus*-induced IFN-γ production and might contribute to the enhanced susceptibility to opportunistic infections.

## Introduction

After major surgery and severe injury patients often experience infectious complications due to the development of immunosuppression [1]. Monocyte deactivation that is characterized by a reduced expression of HLA-DR molecules on the surface and a decreased release of tumor-necrosis factor (TNF) α and Interleukin (IL) 12 in response to microbial stimulation is considered to contribute to immunoparalysis after severe injury [2–4]. The pathomechanisms that lead to post-surgical immunosuppression are only incompletely understood and effective therapies of nosocomial infections especially caused by multi-drug resistant strains are warranted. The successful elimination of bacteria requires the activity of cells of the innate immune system such as macrophages and neutrophilic granulocytes that ingest and eradicate microorganisms and subsequently clear the infection. Upon contact with bacteria, macrophages secrete proinflammatory cytokines such as IL-12, TNF-α, and IL-6. IL-12 activates natural killer (NK) cells for the release of Interferon (IFN) γ that in turn increases the bactericidal activity of macrophages [5]. Thus, the IL-12/IFN-γ axis plays an important role in the defense against bacterial infections.

NK cells belong to the innate immune system and were originally discovered as cytotoxic cells that kill virus-infected host cells or tumor cells. Diverse subsets of human NK cells with different functions can be distinguished according to their relative expression of the adhesion molecule CD56. CD56^dim^ NK cells are highly cytotoxic but are less potent in cytokine secretion [6]. In contrast, CD56^bright^ NK cells have been identified as important regulatory cells in bacterial infections (reviewed in [7]) by their capability to release IFN-γ as mentioned above. NK cell-derived IFN-γ secretion is triggered by IL-12 that is released from accessory cells like monocytes/macrophages or dendritic cells [8,9]. Signaling of IL-12 through the IL-12 receptor (IL-12R) on NK cells leads to the recruitment of the Janus kinase (JAK) 2 and subsequently to the phosphorylation of the transcription factor signal transducer and activator of transcription (STAT) 4 [10]. Phosphorylated STAT4 (pSTAT4) enters the nucleus and induces the transcription of the gene for IFN-γ. In addition to cytokines, direct cell-cell contact of NK cells with accessory cells through the interaction of activating receptors such as Natural killer group 2 member D (NKG2D) on NK cells with their respective ligands on accessory cells modulates the extent of NK cell-derived IFN-γ secretion [11,12].

*Staphylococcus aureus* is a frequent cause of infectious complications after surgery and methicilline resistant *S*. *aureus* strains represent an increasing problem in healthcare worldwide. In this prospective pilot study, we investigated the impact of major surgery on the function of human NK cells in an *in vitro* model of *S*. *aureus* infection. We analyzed the synthesis of IFN-γ in circulating CD56^bright^ NK cells before and at different time points after major visceral surgery in response to *S*. *aureus*. To address the relevance of IL-12 in this infection model, the expression of the IL-12R and downstream phosphorylation of STAT4 in CD56^bright^ NK cells were determined.

## Materials and Methods

### Patients and study design

Patients were admitted to the Department of Visceral Surgery of the University Hospital Essen between March 2011 and August 2012 and were included in the current study. In total 53 patients fulfilled the inclusion criteria of age >18 and elective laparotomy with planed partial or total resection of organs such as liver, intestine, stomach/esophagus. Patients with endoscopic interventions or well established low-risk operations like cholecystectomy were excluded. Other exclusion criteria were: (1) medication with immunosuppressive drugs such as hydrocortisone, (2) preexisting immunological disorders, and (3) any type of infection. Written informed consent of the patients was obtained before the first blood withdrawal. The study was conducted in accordance with the guidelines of the World Medical Association’s Declaration of Helsinki and was approved by the local ethic committee of the University Hospital Essen, Germany.

Venous blood from all patients was withdrawn 1 d before visceral surgery and at different time points (1 to 7 d) after surgery. Heparinized blood was used for the isolation of PBMC. Serum was obtained from clotted blood after centrifugation. PBMC from the first 20 patients who had been included into the study (termed “patients 1”) were used to investigate the kinetics of IFN-γ secretion and the percentage of CD56^bright^ NK cells before and 1, 5, and 7 d after surgery. Thereafter, PBMC from further 33 patients (termed “patients 2”) were isolated before and 1 d after surgery and were analyzed in terms of IL-12 production, IL-12R expression and/or the responsiveness of CD56^bright^ NK cells to IL-12 as indicated in the figure legends. “patients 1” and “patients 2” did not differ in age, gender, hospital stay, cause of surgery, co-morbidity, or pre-medication (Table 1).

**Table 1:** Patients’ characteristics.

	patients 1	patients 2	p value
**Number of patients**	20	33	
Age [y]	61 (53–66)	61 (53–71)	ns
Gender male	14/70	19/58	ns
Hospitalization [d]	13 (10–15)	14 (11–21)	ns
**Cause of surgery**			
Malignom	20/100	30/91	ns
**Resection of**			
Liver	6/30	11/33	ns
Intestine	3/15	4/12	ns
Stomach/Esophagus	4/20	8/25	ns
Others	7/35	10/30	ns
**Co-morbidity**			
Cardiac disease	2/10	8/24	ns
Diabetes	2/10	8/24	ns
**Premedication**			
β-blocker	5/25	8/24	ns
Anticoagulation	5/25	10/30	ns
**Duration of surgery** [min]	187 (132–256)	167 (121–227)	ns
**ICU stay** [d]	1 (0–1)	1 (0–2)	ns
**Infectious complications**	0/0	0/0	ns

Quantitative variables are expressed as median (25^th^-75^th^ interquartile range), qualitative variables are provided as number/% of total. Significant differences between both groups were tested using Mann-Whitney and Fisher’s exact test, respectively.

### Isolation of mononuclear cells and purification of CD56^bright^ NK cells

PBMC were isolated using the standard protocol for Ficoll density gradient centrifugation (Ficoll Paque Premium, GE Health Care, Uppsala, Sweden). After separation, cells were washed twice with endotoxin-tested PBS (Gibco/Invitrogen, Karlsruhe, Germany) and VLE “very low-endotoxin” medium RPMI 1640 (Biochrome, Berlin, Germany) supplemented with 5% fetal calf serum (Sigma Aldrich, St. Louis, USA), respectively.

Primary CD56^bright^ NK cells from PBMC were isolated using the “CD16+/CD56+ NK-cell isolation kit” (Miltenyi, Bergisch Gladbach, Germany) as recommended by the manufacturer. NK cell populations were separated using AutoMACS (Miltenyi). The purity of isolated CD3^-^CD56^bright^ NK cells was > 90% as verified by flow cytometry. The viability of PBMC and isolated NK cells was > 95% as determined by trypan blue exclusion.

### Cell culture

PBMC (4x10^5^ cells/well) and purified CD56^bright^ NK cells (1x10^5^ cells/well) were cultured in VLE RPMI 1640 medium supplemented with 10% autologous serum, 100 U/ml Penicillin, and 100 μg/ml Streptomycin in flat-bottom 96-well plates (BD Falcon, Heidelberg, Germany). All cultures were set up in triplicates in a total volume of 200 μl/well. PBMC were stimulated with 0.05% (v/v) inactivated *S*. *aureus* bacteria (Pansorbin, Merck, Darmstadt, Germany). Unstimulated PBMC served as negative control. Where indicated, recombinant human IL-12 (0.2–0.4 ng/ml as indicated; Biolegend, San Diego, USA) or antibodies (Abs) against human IL-12 (10 μg/ml; clone 24910, R&D, Wiesbaden, Germany) were added to the cultures 1 h prior to stimulation with *S*. *aureus*. Previous experiments have verified that the isotype control Abs against IL-12 (mouse IgG2a isotype control, clone 20102, R&D) did not influence the responsiveness of PBMC to *S*. *aureus* (data not shown). After 19 h, supernatants were harvested and stored at -20°C until use. The cells were used for intracellular staining (see below). Purified CD56^bright^ NK cells were cultured in the presence or absence of 1 ng/ml recombinant IL-12 and 10 ng/ml recombinant IL-18 (MBL International, Woburn, MA). After 19 h, supernatants were harvested and stored at -20°C until use. Cell culture was performed at 37°C in 5% CO_2_ humidified atmosphere.

### Flow cytometry

For the analyses of surface molecule expression, PBMC were incubated with fluorochrome-conjugated Abs against CD3 (clone MEM-57, Fluorescein isothiocyanate-labeled; Immunotools, Friesoythe, Germany), CD56 (clone CMSSB, allophycocyanin-labeled; eBioscience, San Diego, USA), and CD212 (IL-12Rβ1; clone 2.4E6, phycoerythrin-labeled; BD Bioscience). After 12 min. incubation at 4°C, PBMC were washed with CellWash (BD Biosciences, Heidelberg, Germany). Stainings with isotype control Abs were performed to assess the threshold of positive staining for each specific antibody.

To detect intracellular IFN-γ GolgiStop (0.2 μl per well; BD Biosciences) was added to the PBMC at the end of 19 h of culture for further 6 h. Cells of triplicate cultures were pooled and stained with Abs against CD3 and CD56 (see above). After permeabilization of the cells with Cytofix/Cytoperm (BD Biosciences) for 20 min at room temperature, the cells were incubated with phycoerythrin-labeled Abs against IFN-γ (clone 4S.B3, Biolegend) or with the respective isotype control Abs (clone MOPC-21, Biolegend) for 20 min. After washing with permeabilization buffer, the cells were resuspended in CellWash. For intracellular detection of phosphorylated STAT4, cells were stained for CD3 and CD56 after 19 h of culture. Permeabilization and staining with phycoerythrin-labeled anti-pSTAT4 Abs (clone 38/p-STAT4; BD Biosciences) or with the respective isotype control Abs (clone 27–35; BD Biosciences) were carried out using the “FoxP3/Transcription Factor Staining Buffer Set” (eBiosciences) according to the manufacturer’s recommendations. Data acquisition was performed with a FACScalibur (BD Biosciences). Data was analyzed using CellQuest Pro software (BD Biosciences).

### Quantification of cytokines

The concentration of IFN-γ in the cell supernatants from PBMC, and from purified NK cells was determined using enzyme-linked immunosorbent assay (ELISA) (Duoset, R&D) as recommended by the manufacturer. The concentration of IL-12 in the supernatants from PBMC was quantified using a highly sensitive ELISA (Quantikine, R&D). The detection limits of IFN-γ and IL-12 were 15 pg/ml and 0.6 pg/ml, respectively.

### Statistical analysis

Data is presented as scatter plots or as box plots showing the median and interquartile range. Statistical differences were calculated using the non-parametric Wilcoxon signed rank test (comparison of two groups with paired values) or the Friedman test (comparison of multiple groups with paired values) as indicated in the figure legends. Qualitative variables were tested using Fisher’s exact test. A p value < 0.5 was considered as statistically significant. Graph Pad Prism Software (Version 5) was used for all calculations.

## Results

### Major visceral surgery inhibits the response of circulating leukocytes to *S*. *aureus*

**Fig 1:**
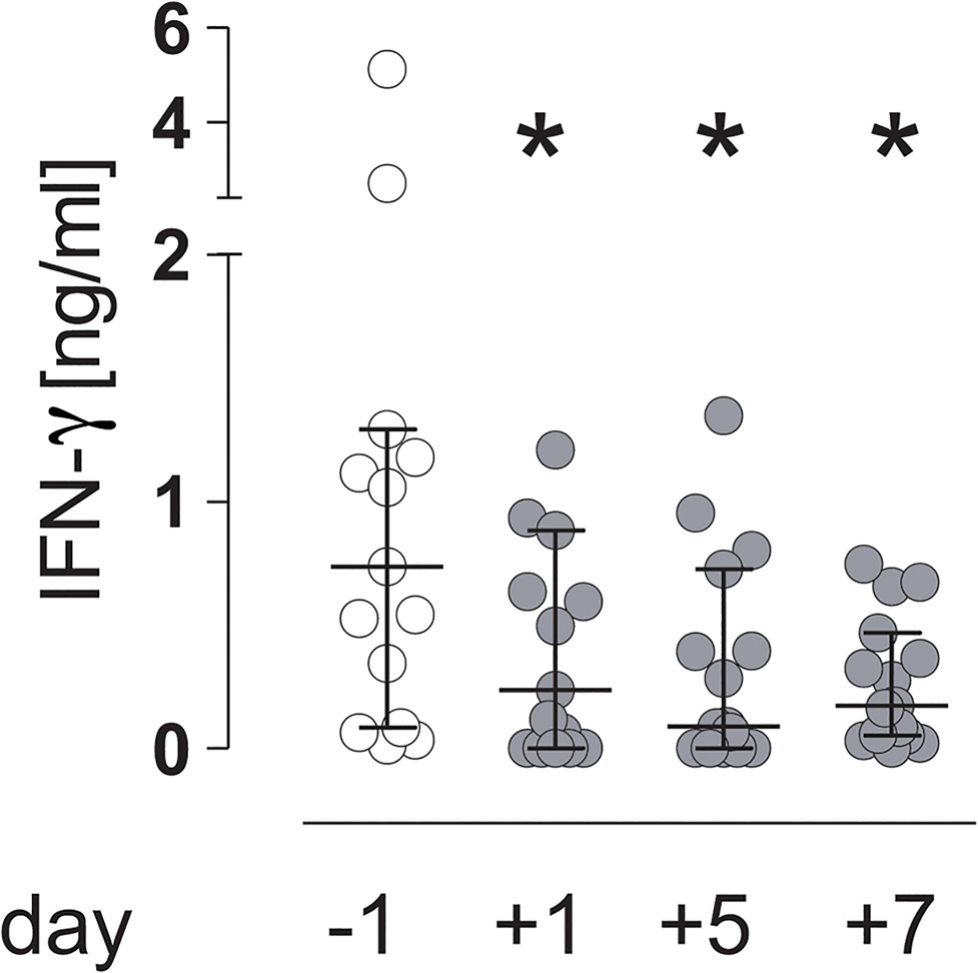
Visceral surgery interferes with the responsiveness to *Staphylococcus aureus*. Peripheral blood from “patients 1” was drawn 1 d before (-1) and 1 d (+1), 5 d (+5), and 7 d (+7) after surgery. PBMC were isolated and stimulated with inactivated *S*. *aureus*. The amount of IFN-γ in the supernatant was quantified. Results are expressed as scatter plot (median, interquartile range). Statistical differences were tested using the Wilcoxon signed rank test. n = 20; *, p<0.05 vs. day -1.

To address the responsiveness of circulating leukocytes to bacteria, PBMC were isolated before and at different time points after major surgery and were stimulated with inactivated *S*. *aureus in vitro*. The content of IFN-γ in the supernatants was determined. Unstimulated PBMC did not produce detectable levels of IFN-γ before and after surgery (data not shown). *S*. *aureus* induced the release of IFN-γ from PBMC obtained before surgery (Fig 1). In contrast, a significantly reduced IFN-γ secretion was observed 1 d after surgery and was maintained for at least 7 d (Fig 1). Thus, major surgical injury causes a reduced responsiveness of circulating leukocytes to *S*. *aureus* in terms of IFN-γ secretion.

### Surgical injury dampens the *S*. *aureus*-induced IFN-γ secretion from circulating CD56^bright^ NK cells

**Fig 2:**
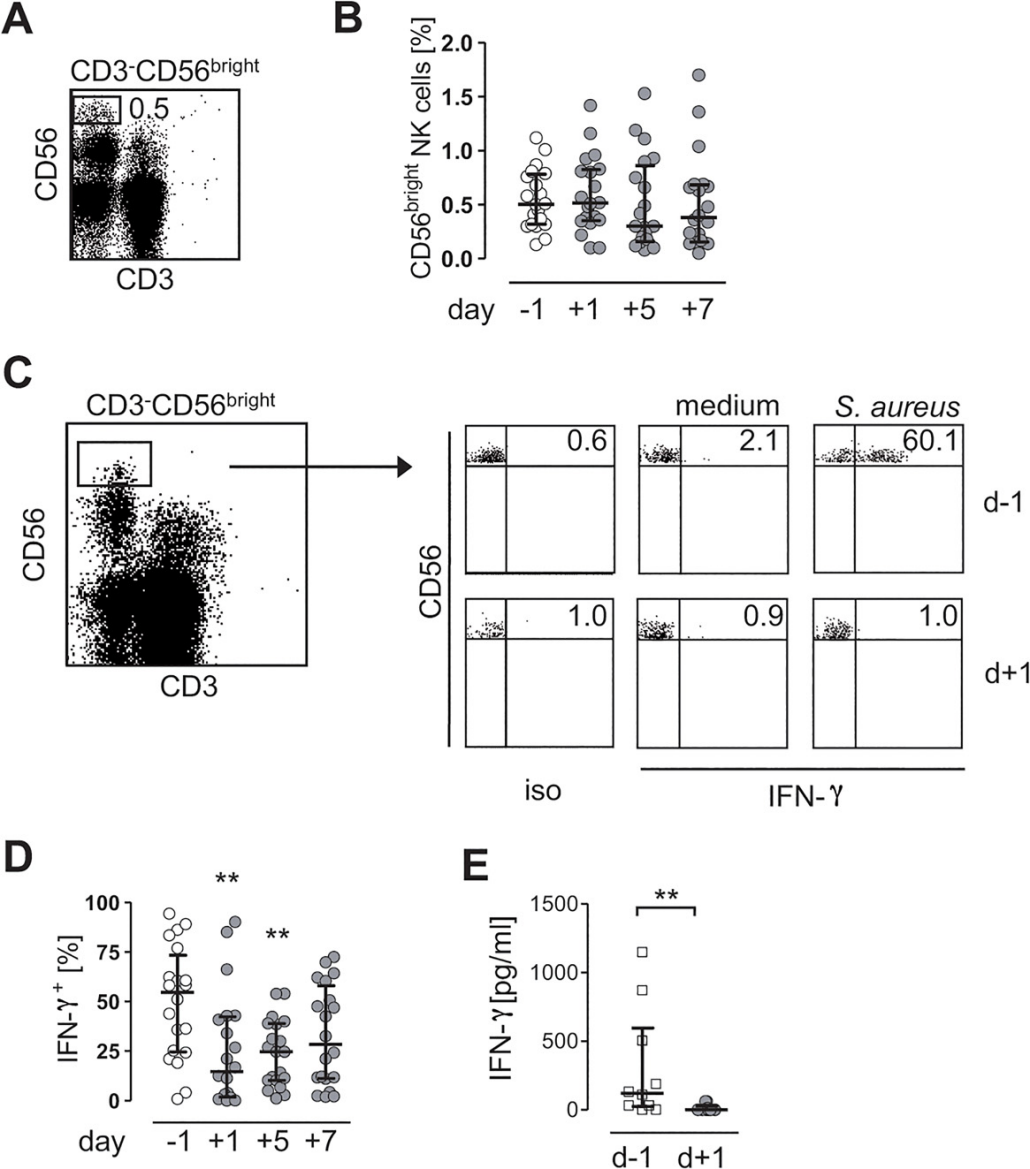
CD56^bright^ NK cells are suppressed in *Staphylococcus aureus*-induced IFN-γ synthesis after invasive surgery. **(A-D)** PBMC were isolated from “patients 1” 24 h before (-1) and 1 d (+1), 5 d (+5), and 7 d (+7) after surgery and were stained against CD3 and CD56. **(A)** Gating strategy of CD3^-^CD56^**bright**^ NK cells among total PBMC. The number indicates the percentage of gated cells in the rectangle. **(B)** Cumulative data on the percentage of gated CD3^-^CD56^**bright**^ NK cells among total PBMC. **(C, D)** PBMC were cultured in the presence of *S*. *aureus* and cells were stained for intracellular IFN-γ. **(C)** Representative dot plots of intracellular IFN-γ expression in gated CD3^-^CD5^**bright**^ NK cells isolated before and 1 d after injury. Numbers indicate the percentage of IFN-γ-positive (IFN-γ^**+**^) cells. **(D)** Cumulative data on the kinetics of IFN-γ^**+**^ CD3^-^CD56^**bright**^ NK cells upon exposure to *S*. *aureus* before and after injuy. **(E)** CD56^**bright**^ NK cells were purified from “patients 2” (n = 11) 1 d before and on day +1 after injury and stimulated with IL-12 and IL-18. The release of IFN-γ into the supernatant was quantified. Results are expressed as scatter plots (median, interquartile range). Statistical differences were tested using the Friedman test (B, D) or the Wilcoxon signed rank test (E). *, p<0.05; **, p<0.01; ***, p<0.001 vs. d-1. iso, isotype control antibodies.

NK cells, especially the CD56^bright^ subset, represent a major source of IFN-γ. We examined whether the reduced release of *S*. *aureus*-induced IFN-γ from PBMC after surgery originated in a decreased percentage of CD56^bright^ NK cells among total PBMC. CD3^-^CD56^bright^ NK cells were gated as shown in Fig 2A. The percentage of CD56^bright^ NK cells among isolated PBMC did not change significantly after surgery. Next, we applied intracellular cytokine staining to determine the synthesis of IFN-γ in individual CD56^bright^ NK cells after stimulation of PBMC with *S*. *aureus*. Before surgery, the stimulation of PBMC with *S*. *aureus* clearly induced the expression of IFN-γ in CD56^bright^ NK cells (Fig 2C and 2D). However, the percentage of IFN-γ-producing CD56^bright^ NK cells strongly decreased within 1 d after surgery and remained reduced for at least further 4 d (Fig 2D). T lymphocytes and natural killer T cells that otherwise represent known sources of IFN-γ did not produce IFN-γ in the context of *S*. *aureus*-stimulated PBMC (See S1 Fig).

To address the question whether the surgery-induced suppression of IFN-γ synthesis was dependent on the presence of accessory cells, CD56^bright^ NK cells were purified and were directly stimulated with IL-12 and IL-18 before determination of the IFN-γ content in the supernatants. Before surgery, CD56^bright^ NK cells secreted IFN-γ into the supernatant, though with interindividual variations (Fig 2E). After surgery, purified CD56^bright^ NK cells were suppressed in their capacity to secrete IFN-γ (Fig 2E). Thus, after major surgery, CD56^bright^ NK cells are impaired in IFN-γ secretion independent of accessory cells.

### CD56^bright^ NK cells only weakly respond to IL-12 after invasive surgery

**Fig 3:**
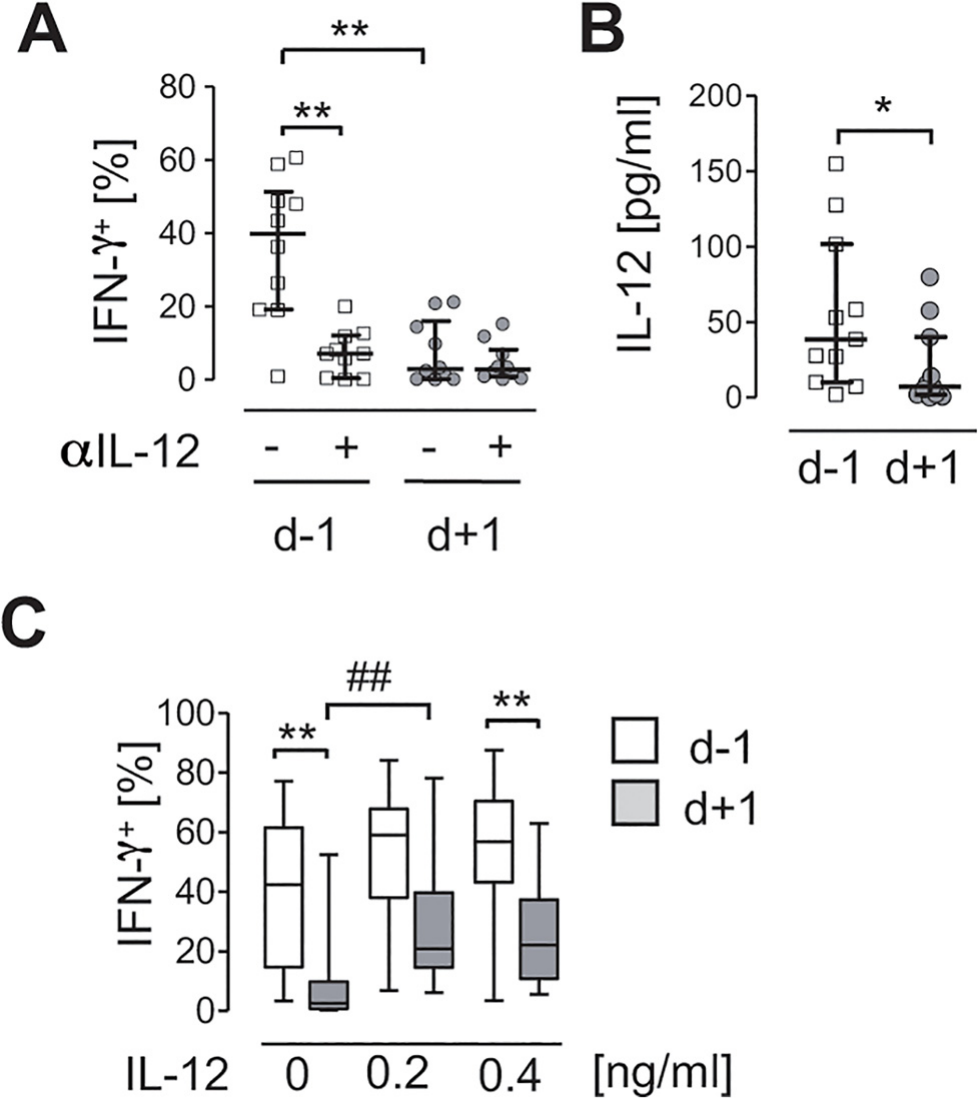
The responsiveness of CD56^bright^ NK cells to IL-12 is reduced after surgery. PBMC from “patients 2” (n = 10) were isolated 24 h before (d-1) and 1 d (d+1) after visceral surgery. **(A)** PBMC were stimulated with *S*. *aureus* in the presence or absence of neutralizing antibodies against IL-12 (αIL-12) and intracellular expression of IFN-γ was analyzed. The percentage of IFN-γ-positive (IFN-γ^**+**^) cells among gated CD3^-^CD56^**bright**^ NK cells was determined as shown in Fig 2C. **(B)** PBMC were stimulated with *S*. *aureus* and the release of IL-12 into the supernatant was quantified (n = 13). Unstimulated PBMC did not secrete IL-12 (data not shown). **(C)** PBMC were stimulated with *S*. *aureus* in the presence or absence of recombinant IL-12 as indicated (n = 17). The percentage of IFN-γ^**+**^ cells among gated CD3^-^CD56^**bright**^ NK cells was determined as performed in Fig 2C. Results are expressed as scatter plots (median, interquartile range) or as box plot (median, interquartile range, range). Statistical differences were tested using the Friedman test (A, C) or the Wilcoxon signed rank test (B). *, p<0.05; ** and ##, p<0.01.

IL-12 derived from accessory cells plays a key role in stimulation of NK cell-derived IFN-γ in diverse infectious diseases. We next investigated whether IL-12 was also relevant for the induction of IFN-γ secretion from CD56^bright^ NK cells upon stimulation of PBMC with *S*. *aureus*. Intracellular staining of IFN-γ demonstrated that neutralization of IL-12 during stimulation of PBMC with *S*. *aureus* strongly impaired the IFN-γ production in CD56^bright^ NK cells before surgery (Fig 3A). Thus, the impaired NK cell-derived IFN-γ production during stimulation of PBMC with *S*. *aureus* after surgery might originate in an inadequate level of IL-12 in the microenvironment. To address this assumption, the content of IL-12 in the supernatant of *S*. *aureus*-stimulated PBMC was quantified before and after surgery. Indeed, PBMC released significantly reduced amounts of IL-12 after surgery (Fig 3B).

Accordingly, we examined whether the supplementation with exogenous IL-12 during stimulation of PBMC with *S*. *aureus* restored the IFN-γ secretion from CD56^bright^ NK cells. The percentage of IFN-γ-producing CD56^bright^ NK cells after surgery significantly increased upon addition of recombinant IL-12 but did not exceed the level of NK cells isolated before surgery (Fig 3C). Thus, the sensitivity of CD56^bright^ NK cells to IL-12 is decreased after major surgery.

### CD56^bright^ NK cells display a decreased IL-12R expression and alteration in STAT4 phosphorylation after surgical injury

**Fig 4:**
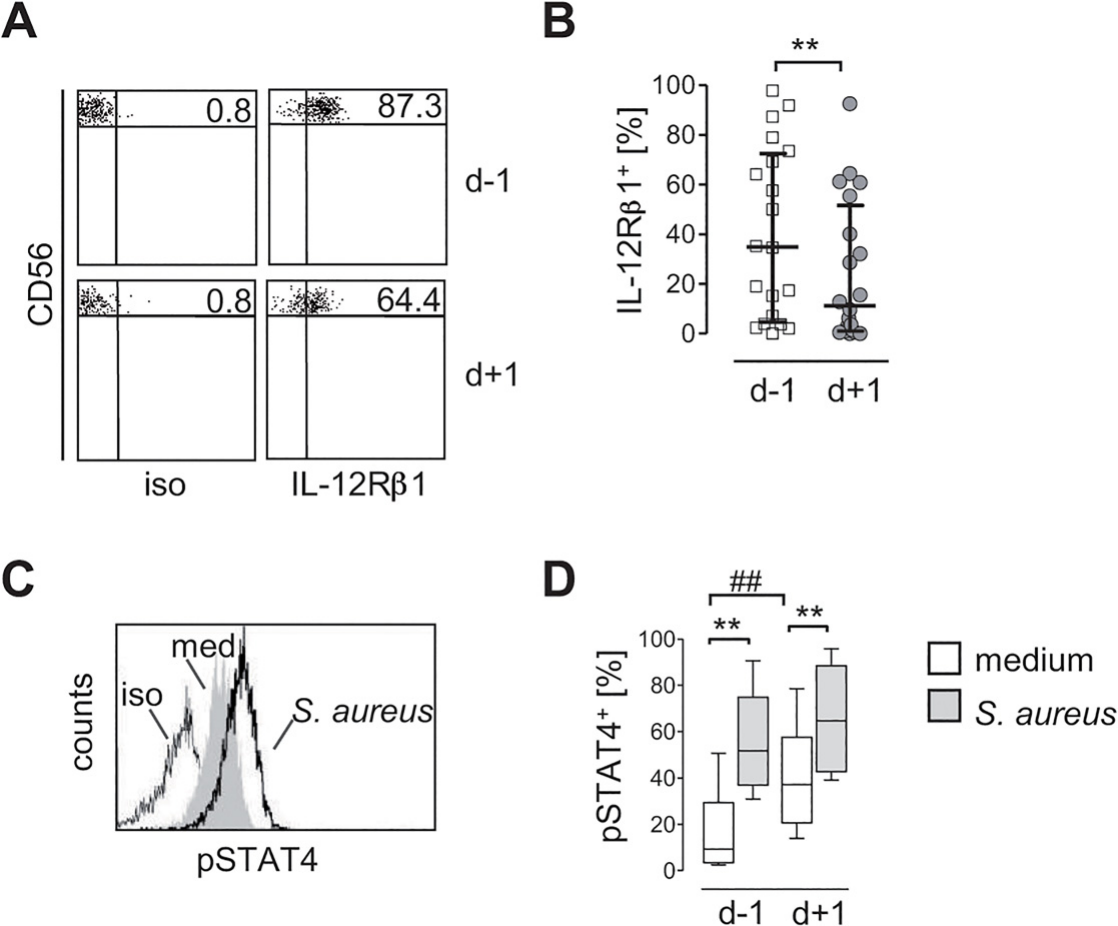
CD56^bright^ NK cells express reduced levels of the IL-12Rβ1 chain but increased levels of phosphorylated STAT4 after injury. PBMC were isolated from “patients 2” 24 h before (d-1) and 1 d (d+1) after injury. PBMC were stained against CD3, CD56, and IL-12Rβ1. CD3^-^CD56^**bright**^ NK cells were gated as shown in Fig 2A. **(A)** Representative dot plot of IL-12Rβ1 expression on gated CD56^**bright**^ NK cells. Numbers indicate the percentage of IL-12Rβ1^**+**^ cells. **(B)** Cumulative data on the percentage of IL-12Rβ1^**+**^ cells among CD56^**bright**^ NK cells before and after injury (n = 20). **(C, D)** PBMC isolated before (d-1) and 1 d (d+1) after injury were cultured in the absence (med) or presence of *S*. *aureus*. Cells were stained against CD3, CD56, and intracellular phosphorylated STAT4 (pSTAT4). **(C)** Representative histogram of pSTAT4 staining in CD56^**bright**^ NK cells analyzed before injury. **(D)** Cumulative data on the percentage of pSTAT4-expressing (pSTAT4^**+**^) cells in gated CD3^-^CD56^**bright**^ NK cells (n = 6). Results are expressed as scatter plot (median, interquartile range) or as box plot (median, interquartile range, range). Statistical differences were tested using the Wilcoxon signed rank test (B) or the Friedman test (D). **, p<0.01; ##, p<0.01. iso, isotype control antibodies.

The finding that CD56^bright^ NK cells after surgery were suppressed in their capacity to release IFN-γ despite optimal stimulation with IL-12 points to a potentially defective responsiveness of these NK cells to IL-12. To address this issue the expression of the IL-12Rβ1 chain on CD56^bright^ NK cells of patients before and after surgery was analyzed (Fig 4A). The percentage of CD56^bright^ NK cells that expressed the IL-12Rβ1 clearly decreased after surgery (Fig 4A and 4B).

Downstream of the IL-12R, we evaluated whether the phosphorylation of STAT4, the key transcription factor for transcription of the *Ifng* gene changed in CD56^bright^ NK cells upon injury. Therefore, PBMC isolated before and after surgery were stimulated with *S*. *aureus* or were left untreated. Since the number of CD56^bright^ NK cells that could be isolated from PBMC of the patients was too low to perform Western Blot analyses, the level of phosphorylated STAT4 was determined by flow cytometry (a representative histogram is shown in Fig 4C). Before surgery, the stimulation of PBMC with *S*. *aureus* led to a more than 5-fold increase in STAT4 phosphorylation in CD56^bright^ NK cells (Fig 4D). Remarkably, even in the absence of *S*. *aureus* CD56^bright^ NK cells that had been isolated after surgery displayed a 4-fold enhanced level of pSTAT in comparison to CD56^bright^ NK cells obtained before surgery (Fig 4D). After surgery, the stimulation of PBMC with *S*. *aureus* further increased the level of pSTAT4 in CD56^bright^ NK cells by 1.8-fold (Fig 4D). Thus, the impaired IFN-γ synthesis of CD56^bright^ NK cells after surgery is associated with a decline in IL-12R expression but not in STAT4 phosphorylation.

## Discussion

In the present pilot study we provide novel insights into the function of NK cells after surgery. Major visceral surgery suppressed the capacity of circulating CD56^bright^ NK cells to secrete IFN-γ in the environment of leukocytes stimulated with *S*. *aureus*. The inhibition of CD56^bright^ NK cells was associated with a reduced expression of the IL-12Rβ1 chain. Accordingly, after surgery CD56^bright^ NK cells only weakly responded to IL-12 with the synthesis of IFN-γ. However, *S*. *aureus*-induced STAT4 phosphorylation in CD56^bright^ NK cells was not reduced.

In line with previous reports we observed that PBMC secreted less IFN-γ in response to *S*. *aureus* after severe injury [13,14]. NK cells are considered to represent the major source of IFN-γ among PBMC after stimulation with *S*. *aureus* [14,15]. A reduction of the number of circulating CD56^bright^ NK cells has been observed after cardiac surgery [14] and might have been a trivial explanation for the reduced IFN-γ release from PBMC after visceral surgery. However, the unchanged percentage of CD56^bright^ NK cells in our study rather pointed to a surgery-induced alteration of NK cell function.

Using the approach of intracellular cytokine staining we now show here for the first time that surgical injury dampens the capacity of individual circulating CD56^bright^ NK cells to produce IFN-γ in the environment of PBMC exposed to *S*. *aureus*. NK cells themselves do not respond to *S*. *aureus* but require the cooperation with accessory cells like monocytes and dendritic cells though there exists conflicting data on the nature of such accessory cells [14–16].

We identified IL-12, a product of accessory cells, as the key cytokine that was necessary for the synthesis of IFN-γ in CD56^bright^ NK cells upon stimulation of PBMC with *S*. *aureus*. PBMC were impaired in the *S*. *aureus*-induced release of IL-12 after injury possibly due to the deactivation of monocytes or to the loss of dendritic cells in peripheral blood, both events being known consequences of severe tissue damage in humans [4,13,14]. This observation led to the assumption that the failure of accessory cells to secrete IL-12 might be responsible for the suppression of CD56^bright^ NK cell-derived IFN-γ synthesis. Indeed, the formation of *S*. *aureus*-induced IFN-γ in CD56^bright^ NK cells after surgery increased in the presence of additional recombinant IL-12 but still remained inferior to CD56^bright^ NK cell obtained before surgery. Thus, the reduced *S*. *aureus*-induced secretion of IL-12 may only partly explain the impaired IFN-γ synthesis of CD56^bright^ NK cells after surgery. The regulatory cytokine IL-10 inhibits NK cell-derived IFN-γ synthesis and is known to be released after injury [17,18]. In our study, neutralization of IL-10 during stimulation of PBMC with *S*. *aureus* did not increase the formation of IFN-γ after surgery (See S2 Fig). Therefore, we assume that the suppressed responsiveness of NK cells after major surgery was largely independent of accessory cells and of an imbalance of IL-12 and IL-10 in the microenvironment but rather points to a direct surgery-induced modulation of CD56^bright^ NK cells.

Surface molecules that otherwise indicate the activation of NK cells and in part contribute to IFN-γ synthesis such as NKG2D, NKp46, or CD62L [11,19,20] remained unchanged after surgery (See S3 Fig). In contrast, CD56^bright^ NK cells expressed reduced levels of the IL-12Rβ1 chain. We suggest that this diminished IL-12Rβ1 expression contributes to a reduced responsiveness of CD56^bright^ NK cells to IL-12 after surgery and consequently might prevent adequate production of IFN-γ even in the presence of sufficient amounts of IL-12. This assumption is supported by the finding that purified CD56^bright^ NK cells isolated after injury were incompetent in IFN-γ secretion despite stimulation with a sufficient amount of recombinant IL-12.

Unexpectedly, CD56^bright^ NK cells expressed higher base-line levels of the downstream transcription factor pSTAT4 after surgery although the expression of the IL-12Rβ1 was reduced. Considering the importance of pSTAT4 in transcription of the *Ifng* gene this finding is in marked contrast to the suppressed capacity of CD56^bright^ NK cells for IL-12-induced IFN-γ secretion. The question arises which signal(s) induced STAT4 phosphorylation after surgical injury and why pSTAT4 did not result in IFN-γ production. In addition to the IL-12R-associated kinase JAK2, phosphorylation of STAT4 may be mediated by the mitogen-activated protein kinase p38 that is involved in Toll-like receptor (TLR) signaling [21]. Endogenous danger molecules and TLR agonists such as high mobility group box 1 protein, S100A8/A9, and heat shock proteins are released upon tissue injury-induced cell necrosis [22–24] and might be involved in STAT4 phosphorylation via TLR/p38 signaling in circulating CD56^bright^ NK cells after visceral surgery. Moreover, the suppressed IFN-γ production that occurs despite existing STAT4 phosphorylation might be caused by an inappropriate function of other transcription factors like T-bet, a known regulator of pSTAT4 binding to the *Ifng* promoter and of subsequent transcription [25]. So far, we cannot provide any evidence that confirms the above mentioned speculations on the association of STAT4 phosphorylation and impaired IFN-γ production in CD56^bright^ NK cells after surgery but adequate studies to answer this question are in progress.

In a murine two-hit model the depletion of NK cells shortly after traumatic injury increases the survival rate after subsequent induction of polymicrobial sepsis [26]. Moreover, we recently showed that immunosuppression after traumatic injury does not develop in the absence of NK cells [27]. NK cells are recruited from the circulation to the site of infection where they contribute to the coordination of the immune response [28,29]. Altogether, these findings led to our hypothesis that, after surgical injury, CD56^bright^ NK cells recruited from the circulation fail to support the defense against invading pathogens due to inefficient IFN-γ synthesis. This hypothesis might provide an explanation for the fact that surgical injury and severe trauma enhance the risk for life-threatening opportunistic infections frequently with *S*. *aureus* [30]. The limitation of the present study was that the number of patients was too low to verify an association between the suppression of NK cells and the occurrence of infectious complications. Souza-Fonseca-Guimaraes et al. recently reported a suppressed IFN-γ synthesis of NK cells in patients with sepsis and SIRS and, thereby, support the link between NK cell suppression and critical illness though the role of IL-12 was not addressed [31]. In addition to the IFN-γ production, as we show here, surgery interferes with the other key function of NK cells: the cytotoxicity to tumor cells [32]. Further studies are required to elucidate whether surgery disturbs the immune regulatory as well as the cytotoxic function of NK cells through similar mechanisms.

In summary, our study shows that invasive surgical injury induces an unresponsiveness of circulating human CD56^bright^ NK cells to IL-12 that is associated with a decrease in IL-12R expression but not STAT4 activation. Accordingly, CD56^bright^ NK cells are impaired in IFN-γ secretion and might promote the enhanced susceptibility to bacterial infections after invasive surgery. Such patients might benefit from the development of therapies that restore the capacity of NK cells to respond to IL-12 with the appropriate secretion of IFN-γ.

## Supporting Information

S1 Fig IFN-γ expression in natural killer T cells and T cells upon exposure to *Staphylococcus aureus* after invasive surgery. (PDF)

S2 Fig Neutralization of IL-10 during stimulation with *S*. *aureus*. (PDF)

S3 Fig Expression of NKG2D, CD62L, and NKp46 on CD56^hi^ NK cells after invasive surgery. (PDF)
