# Supplementary material for: Invasive Surgery Impairs the Regulatory Function of Human CD56bright Natural Killer Cells in Response to Staphylococcus aureus. Suppression of Interferon-γ Synthesis
Source: PLoS One. 2015 Jun 19;10(6):e0130155. doi: 10.1371/journal.pone.0130155 (PMC4474941; doi:10.1371/journal.pone.0130155)
Supplement: S1 Fig — (PDF) [file pone.0130155.s001.pdf]

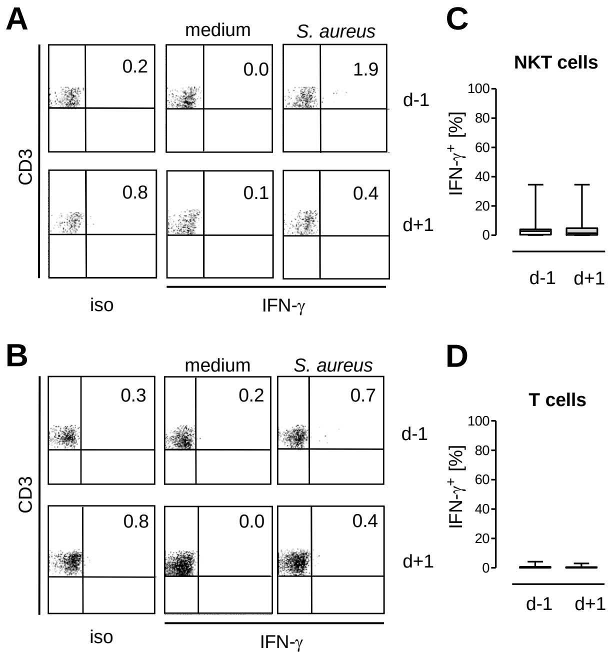

S1 Figure. IFN-γ expression in natural killer T cells and T cells upon exposure to *Staphylococcus aureus* after invasive surgery. Peripheral blood from "patients 2" (n=20) was drawn 24 h before (d-1) and 1 d after injury (d+1). PBMC were isolated and cultured in the presence or absence of *S. aureus*. After addition of GolgiStop the cells were stained against CD3, CD56, and intracellular IFN-γ. Representative dot plots of intracellular IFN-γ expression in gated **(A)** $CD3^{+}CD56^{+}$ natural killer T (NKT) cells and **(B)** $CD3^{+}CD56^{-}$ T cells. Numbers indicate the percentage of IFN-γ-positive (IFN-γ^{+}) cells. Cumulative data on the percentage of IFN-γ-positive **(C)** NKT cells and **(D)** T cells upon exposure to *S. aureus*. Results are expressed as box plots showing the median, interquartile range, and range. Statistical differences were tested using the Wilcoxon signed rank test. iso, isotype control antibodies
