# Supplementary material for: Invasive Surgery Impairs the Regulatory Function of Human CD56bright Natural Killer Cells in Response to Staphylococcus aureus. Suppression of Interferon-γ Synthesis
Source: PLoS One. 2015 Jun 19;10(6):e0130155. doi: 10.1371/journal.pone.0130155 (PMC4474941; doi:10.1371/journal.pone.0130155)
Supplement: S2 Fig — (PDF) [file pone.0130155.s002.pdf]

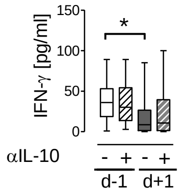

S2 Figure. Neutralization of IL-10 during stimulation with *S. aureus*. Peripheral blood from “patients 1” (n=10) was drawn 24 h (d-1) before and 1 d after injury (d+1). PBMC were stimulated with *S. aureus* in the presence or absence of 10 µg/ml neutralizing monoclonal antibodies against IL-10 (αIL-10). The release of IFN-γ into the supernatant was determined by ELISA. Statistical differences were tested using the Friedman test followed by Dunn’s multiple comparison test. *, $p<0.05$
