# Supplementary material for: Invasive Surgery Impairs the Regulatory Function of Human CD56bright Natural Killer Cells in Response to Staphylococcus aureus. Suppression of Interferon-γ Synthesis
Source: PLoS One. 2015 Jun 19;10(6):e0130155. doi: 10.1371/journal.pone.0130155 (PMC4474941; doi:10.1371/journal.pone.0130155)
Supplement: S3 Fig — (PDF) [file pone.0130155.s003.pdf]

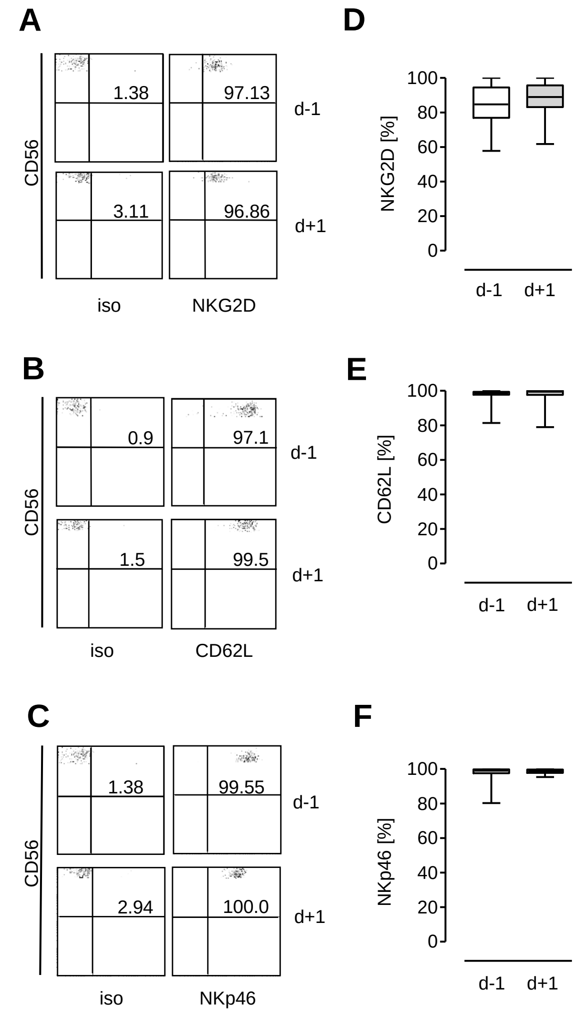

S3 Figure. Expression of NKG2D, CD62L, and NKp46 on CD56^{hi} NK cells after invasive surgery. Peripheral blood from “patients 2” (n=18) was drawn 24 h before (d-1) and 1 d after injury (d+1). PBMC were stained with fluorochrome-conjugated Abs against CD3, CD56, and NKG2D, CD62L, NKp46, or the corresponding isotype control Abs (iso). Representative dot plots of NKG2D **(A)**, CD62L **(B)**, NKp46 **(C)** on gated CD3^{-}CD56^{hi} NK cells. Numbers indicate the percentage of positive cells. Cumulative data on the expression of NKG2A **(D)**, CD62L **(E)**, and NKp46 **(F)** on CD56^{hi} NK cells from all patients. Data are expressed as box plots showing the median, interquartile range, and range. Statistical differences were tested using the Wilcoxon signed rank test.
